# Matcha Green Tea Improves Cafeteria-Diet-Induced NAFLD by Modulating the Gut Microbiota in Rats

**DOI:** 10.3390/nu17193051

**Published:** 2025-09-24

**Authors:** Ho-Ching Chong, Shu-Ting Tang, Yu-Chieh Tseng, Suh-Ching Yang, Yasuo Watanabe, Shizuo Yamada, Yu-Chen S. H. Yang, Ya-Ling Chen

**Affiliations:** 1 School of Nutrition and Health Sciences, Taipei Medical University, Taipei 11031, Taiwan; ma07109019@tmu.edu.tw (H.-C.C.); ma07110021@tmu.edu.tw (S.-T.T.); ma07111006@tmu.edu.tw (Y.-C.T.); sokei@tmu.edu.tw (S.-C.Y.); 2 General Health Medical Center, Yokohama University of Pharmacy, Yokohama 245-0066, Japan; yasuwat@yok.hamayaku.ac.jp; 3 Center for Pharma-Food Research, Graduate School of Pharmaceutical Sciences, University of Shizuoka, Yokohama 422-8526, Japan; yamada@u-shizuoka-ken.ac.jp; 4 Joint Biobank, Office of Human Research, Taipei Medical University, Taipei 11031, Taiwan; can_0131@tmu.edu.tw

**Keywords:** cafeteria diet, matcha, obesity, low-grade inflammation, dysbiosis, rat model

## Abstract

**Background:** The aim of this study was to investigate the effects of matcha on lipid metabolism, insulin resistance (IR), inflammation, and gut dysbiosis in non-alcoholic fatty liver disease (NAFLD) induced by a cafeteria diet. **Methods:** Forty-eight 7-week-old male Wistar rats were divided into six groups (*n* = 8), including a control group (C), C + 0.2% matcha group (C + 0.2%), C + 1% matcha group (C + 1%), cafeteria group (Caf), Caf + 0.2% matcha group (Caf + 0.2%), and Caf + 1% matcha group (Caf + 1%). All rats were sacrificed at the end of the 12th week of the experiment. A one-way analysis of variance (ANOVA), followed by a Fisher’s post hoc test, was used to determine the significant differences among each of the groups. **Results:** The results indicated that plasma experiment triglycerides (TGs) significantly increased in the Caf group compared to the C group, and significantly decreased TG levels were found in the Caf + 1% group compared to the Caf group. In addition, the liver total cholesterol and TG had significantly increased in the Caf group, while the 0.2% Matcha intervention can mitigate hepatic lipid accumulation. Blood sugar, serum insulin, the homeostasis model assessment of IR (HOMA-IR), and plasma leptin significantly increased in the Caf group and were significantly lower in the Caf + 0.2% and Caf + 1% groups. Hepatic cytokines significantly increased in the Caf group, while, on the other hand, significantly lower concentrations were found in the Caf + 1% group. In addition, beneficial bacteria including *Akkermansia*, *Faecalibacterium*, and *Parabacteroides* increased after matcha supplementation. **Conclusions:** These results suggested that 12 weeks of a cafeteria diet can induce abnormal lipid metabolism, IR, liver inflammation, and an altered gut microbiotic composition, while both the 0.2% and 1% matcha interventions might regulate obesity, lipid accumulation, IR, and inflammatory responses, and help maintain a healthier gut microbiota, which may then ameliorate the development of NAFLD.

## 1. Introduction

Non-alcoholic fatty liver disease (NAFLD) is a multifactorial chronic liver disease which is usually caused by an unhealthy lifestyle, especially bad eating habits and a lack of exercise. There are 30% NAFLD patients among the world’s population, and about 36.1% of the people in Taiwan are NAFLD patients; the prevalence of NAFLD is still increasing globally [1,2]. NAFLD usually coexists with obesity, and common pathophysiological abnormalities underlie NAFLD, obesity, and type 2 diabetes mellitus (T2DM) [3]. NAFLD is characterized by a >5% hepatic lipid accumulation in hepatocytes without a history of alcohol consumption [4], and it can be divided into four processes: steatosis, non-alcoholic steatohepatitis (NASH), cirrhosis, and hepatocellular carcinoma (HCC) [5].

The multi-hit hypothesis is commonly used to explain the complex mechanism of NAFLD, such as abnormal lipid metabolism, insulin resistance (IR), inflammation, and gut dysbiosis. IR is regarded as the “first hit” that leads to an increased uptake and the synthesis of free fatty acids (FFAs) stored as triglycerides (TGs), resulting in simple steatosis [6]. Increasing β-oxidation under steatosis produces reactive oxygen species (ROS) which cause lipid peroxidation and oxidative stress [7]. Oxidative stress triggers the release of proinflammatory cytokines, including tumor necrosis factor (TNF)-α, interleukin (IL)-1β, and IL-6. The rise in cytokine levels causes liver inflammation and begins a vicious cycle of the stimulation of cytokine release through the Toll-like receptor 4 (TLR4)/nuclear factor kappa-light-chain-enhancer of activated B (NF-κB) pathway. These mechanisms accelerate the process of NAFLD [8]. Adipokines, including adiponectin and leptin which are released from adipose tissues, play key roles in NAFLD [9]. Adiponectin has anti-inflammatory, antiobesity, and IR-regulatory properties [10]. Leptin is an adipokine that mainly functions in the hypothalamus, and it can inhibit the appetite and increase the energy expenditure [9]. However, a long-term, high level of leptin can cause leptin resistance, which causes NAFLD individuals to exhibit a low adiponectin level and high leptin level. An abnormal adipokine level can cause an increase in appetite and a reduction in energy expenditure [10]. The alteration in the gut microbial population is one of the most common risk factors for NAFLD [11]. Furthermore, patients with NAFLD showed the overgrowth of small intestinal bacteria, which can lead to gut dysbiosis [12].

The cafeteria diet is a popular animal diet for NAFLD models especially in Western countries; it comprises high-fat, high-sodium, high-caloric, low-dietary-fiber junk food and processed foods. Compared to a traditional high-fat diet, the cafeteria diet can comprehensively increase macronutrients, and it is quite similar to the common Western diet nowadays [13]. It was reported to stimulate the voluntary hyperphagia of rodent animals and cause rapid weight gain and insulin intolerance [14]. Moreover, the cafeteria diet was reported to cause liver steatosis and NASH in animal models [15].

Japanese matcha is a kind of powdered green tea product, which contains high contents of flavonoid and polyphenol compounds including catechin and caffeine due to a “shading” process initiated 20~25 days before harvest [16]. Based on previous research, matcha has anti-inflammatory, antiobesity, and antioxidative properties [17,18].

However, there is lack of studies related to the function of matcha green tea against cafeteria-diet-induced NAFLD in a rat model. In this study, we investigated the potential of matcha green tea in protecting against lipid accumulation, IR, inflammation, and gut dysbiosis caused by the consumption of a cafeteria diet composed of common Taiwanese junk foods and processed foods in a rat model. We hypothesized that supplementation with 0.2% and 1% matcha powder in animal feed would attenuate the lipid accumulation, insulin resistance, and inflammatory responses induced by a cafeteria diet, and that these effects might be mediated through the modulation of the gut microbiota in a rat model.

## 2. Materials and Methods

### 2.1. Matcha Powder

Matcha powder was obtained from Kawane Matcha (Shizuoka, Japan). As shown in Table 1, active compounds in matcha include gallocatechin (GC), epicatechin (EC), epicatechin gallate (ECG), epigallocatechin (EGC), and epigallocatechin gallate (EGCG), and these were quantified by high-performance liquid chromatography (HPLC), while the total polyphenol content was quantified by ultraviolet-visible spectrophotometry conducted by SGS Taiwan (New Taipei City, Taiwan).

### 2.2. Animals and Diets

After 1 week of acclimatization, 48 male Wistar rats (7 weeks old, BioLasco Taiwan, Yilan, Taiwan) were randomly divided into 6 groups with 8 rats in each group (*n* = 8): a control group (C), control + 0.2% matcha group (C + 0.2%), control + 1% matcha group (C + 1%), cafeteria group (Caf), cafeteria + 0.2% matcha group (Caf + 0.2%), and cafeteria + 1% matcha group (Caf + 1%). All rats were housed individually.

#### Study Design

After one-week acclimatization, all rats were fed their specific diets and water ad libitum for 12 weeks. The C group was given a standard rodent diet (LabDiet 5001 Diet, PMI Nutrition International, St. Louis, MO, USA) which provided 3.36 kcal/g, while the Caf groups were given a cafeteria diet according to our previous study [19], which contained 4.34 kcal/g, with macronutrient energy percentages of 46% carbohydrates, 38% fats, and 16.0% proteins. The C + 0.2% and Caf + 0.2% diets contained 0.2% (g/g) of matcha powder, which was equal to half a cup of matcha tea (containing 1.2 g of matcha powder) per day for humans, while the C + 1% and Caf + 1% diets contained 1% (g/g), which was equal to about three cups of matcha tea (containing 6 g of matcha powder) per day for humans.

Diet consumption was measured every 2 days, while body weights (BWs) were measured every week. Rats were anesthetized and sacrificed at the end of the 12-week experiment. Blood samples were collected and centrifuged (at 1800× *g* and 4 °C for 15 min) and were stored at −80 °C until being assayed. The liver was rapidly excised and stored at −80 °C for further analysis. All procedures were approved by the Institutional Animal Care and Use Committee of Taipei Medical University (identification code: LAC-2021-0170).

### 2.3. Blood Biochemical Analyses

Plasma aspartate aminotransferase (AST), alanine aminotransferase (ALT), total cholesterol (TC), total triglycerides (TGs), low-density lipoprotein cholesterol (LDL-C), and high-density lipoprotein cholesterol (HDL-C) were detected with the ADVIA^®^ Chemistry XPT System (Siemens Healthcare Diagnostics, Eschborn, Germany).

### 2.4. Liver TC and TG Concentrations

To measure liver TC concentrations, 0.01 g of liver tissue was added to 200 μL of solvent containing chloroform: isopropanol: nonyl phenoxypolyethoxylethanol (NP-40) (7:11:0.1). The homogenized solution was centrifuged to separate the supernatant (at 15,000× *g* and 4 °C for 10 min). The supernatant was air-dried for 50 min and placed in a vacuum for 30 min to remove all of the solvent. Then, 200 μL of the assay diluent was added to dissolve the dried lipid, and it was fully vortexed. The dissolved solutions were stored at −80 °C for further analysis. To measure liver TG concentrations, 0.1 g of liver tissue was homogenized with 500 μL NP-40 which contained protease inhibitors, and then centrifuged at 10^4^× *g* and 4 °C for 30 min. The supernatant was collected and stored at −80 °C for further analysis. Liver TC and TG concentrations were analyzed with a cholesterol colorimetric assay kit (Cell Biolabs, San Diego, CA, USA) and a TG colorimetric assay kit (Cayman Chemical, Ann Arbor, MI, USA).

### 2.5. Liver Histopathological Analysis

The liver caudate lobe was fixed in a 10% formaldehyde solution. Hematoxylin and eosin (H&E) stain was evaluated by a veterinarian. Tissue images were captured at 200× magnification. Macrovascular steatosis, microvascular steatosis, hypertrophy, and the number of inflammatory foci were separately scored and graded based on a previous study by Liang [20]. Scores were added up to calculate the NAFLD score.

### 2.6. Insulin Resistance (IR)

Serum insulin levels were assayed with a Rat Insulin ELISA (enzyme-linked immunosorbent assay) kit (Mercodia, Uppsala, Sweden). The fasting blood glucose level was detected with the Glucose Monitoring System (Abbott Diabetes Care, Oyl, UK). HOMA-IR was calculated using the following formula:

HOMA-IR = (Fasting blood glucose (mg/dL) × Fasting insulin (mIU/L))/405.

### 2.7. Liver Cytokine Levels

The extraction method of liver tissues was based on Chen et al.’s study [21], in which 0.5 g of liver tissues was homogenized in 1.5 mL of ice-cold buffer. The buffer contained 50 mM Tris (pH 7.2), 1% Triton X-100, 150 mM NaCl, and 0.1% protease inhibitor (PI) (HYK0010, MedChemExpress, Monmouth Junction, NJ, USA). Then, the homogenized solution was centrifuged (at 664× *g* and 4 °C for 15 min), and the supernatant was collected. Hepatic TNF-α, IL-1β, IL-6, and IL-10 levels were determined with ELISA kits, including rat TNF-α (BioLegend Systems, San Diego, CA, USA), IL-1β (Rat IL-1, R&D Systems, Minneapolis, MN, USA), IL-6 (Rat IL-6, R&D Systems), and IL-10 (Rat IL-10, R&D Systems). All cytokines were read at an absorbance of 450 nm with a microplate reader (Molecular Devices, Sunnyvale, CA, USA).

### 2.8. Plasma Adipokine Concentrations

Plasma leptin and adiponectin levels were determined with Leptin Mouse/Rat ELISA (Biovendor, Brno, Czech Republic) and Rat Adiponectin (Assay Max™, St. Charles, LA, USA) commercial ELISA kits. Both adipokines were read at an absorbance of 450 nm with a microplate reader (Molecular Devices). The adiponectin/leptin ratio was calculated after the above analysis.

### 2.9. Epididymal White Adipose Tissue (eWAT) Histopathological Analysis

eWATs were fixed in a 10% formaldehyde solution. H&E staining was evaluated by a veterinarian. Images of the tissues were captured at 200× magnification.

### 2.10. Assessment of the Fecal Microbiotic Composition

Fresh feces was collected at the end of the study and stored at −80 °C for analysis. The 16S ribosomal (r)RNA Next Generation Sequencing (NGS) system was performed to analyze fecal microbiotic composition as described by Yang et al. [22]

### 2.11. Statistical Analysis

All data are expressed as the mean ± standard deviation (SD). GraphPad Prism vers. 8.0.1 (GraphPad Software, San Diego, CA, USA) was used for the statistical analysis. A one-way analysis of variance (ANOVA) followed by Fisher’s post hoc test was used to determine significant differences among the C, C + 0.2%, C + 1%, Caf, Caf + 0.2%, and Caf + 1% groups. Statistical significance was assigned at *p* < 0.05.

## 3. Results

### 3.1. Effect of Matcha on Food Intake, Energy Intake, and BWs

**Figure 1:**
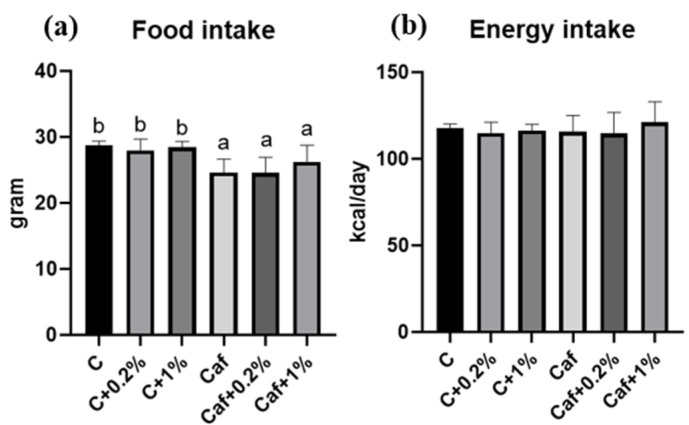
Food intake and energy intake in each group. (**a**) Food intake and (**b**) energy intake. Values are presented as the mean ± standard deviation (*n* = 8). ^a,b^ Different letters indicate a significant difference between groups at *p* < 0.05 by a one-way ANOVA with Fisher’s post hoc test. C, control diet; C + 0.2%, control diet + 0.2% matcha; C + 1%, control diet + 1% matcha; Caf, cafeteria diet; Caf + 0.2%, cafeteria diet + 0.2% matcha; Caf + 1%, cafeteria diet + 1% matcha.

The related results are shown in Figure 1a,b. First, the food intake levels of the Caf groups were significantly lower than that of the C group (*p* < 0.05), but there was no significant difference among the Ca, Caf + 0.2%, and Caf + 1% groups. Second, the energy intake levels showed no significant differences among all groups.

On the other hand, BWs of the Caf groups were significantly higher than those of the C group beginning from the fourth week (*p* < 0.05), while there were no significant differences among the Caf, Caf + 0.2%, and Caf + 1% groups (Table 2).

### 3.2. Effects of Matcha on Liver, eWAT, and Perirenal (p)WAT Weights

**Figure 2:**
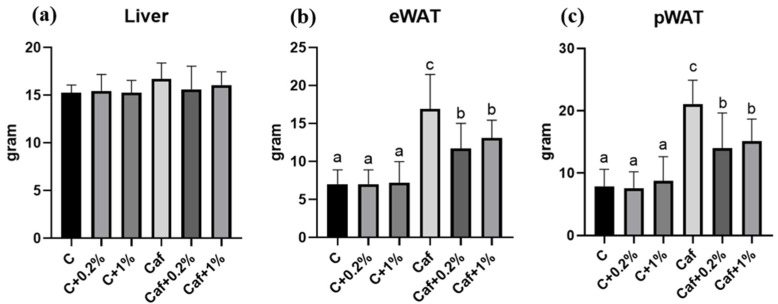
Effects of matcha on (**a**) liver weight, (**b**) epididymal white adipose tissue (eWAT) weight, and (**c**) perirenal (p)WAT weight in rats with cafeteria diet feeding. Values are presented as the mean ± standard deviation (*n* = 8). ^a,b,c^ Different letters indicate a significant difference between groups at *p* < 0.05 by a one-way ANOVA with Fisher’s post hoc test. C, control diet; C + 0.2%, control diet + 0.2% matcha; C + 1%, control diet + 1% matcha; Caf, cafeteria diet; Caf + 0.2%, cafeteria diet + 0.2% matcha; Caf + 1%, cafeteria diet + 1% matcha.

As for liver weights, there were no significant differences among the six groups (Figure 2a). Both the eWAT and pWAT weight results revealed that the Caf groups had significantly higher weights than the C group (*p* < 0.05). The matcha and Caf intervention groups had significantly reduced eWAT and pWAT weights compared to the Caf groups (*p* < 0.05) (Figure 2b,c).

### 3.3. Effect of Matcha on Liver Function and the Lipid Profile

**Figure 3:**
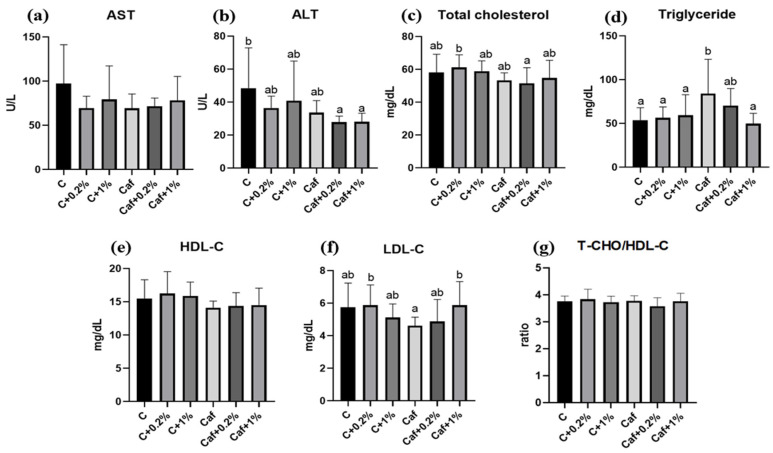
Effects of matcha on liver function and the lipid profile in rats fed a cafeteria diet. (**a**) Aspartate transaminase (AST), (**b**) alanine transaminase (ALT), (**c**) total cholesterol (T-CHO), (**d**) triglycerides, (**e**) high-density lipoprotein cholesterol (HDL-C), (**f**) low-density lipoprotein cholesterol (LDL-C), and (**g**) T-CHO/HDL-C ratio. Values are presented as the mean ± standard deviation (*n* = 8). ^a,b^ Different letters indicate a significant difference between groups at *p* < 0.05 by a one-way ANOVA with Fisher’s post hoc test. C, control diet; C + 0.2%, control diet + 0.2% matcha; C + 1%, control diet + 1% matcha; Caf, cafeteria diet; Caf + 0.2%, cafeteria diet + 0.2% matcha; Caf + 1%, cafeteria diet + 1% matcha.

As shown in Figure 3, there were no significant differences in plasma AST activity among the six groups. As for the ALT activities, there was no significant difference between the Caf and C groups. There were no significant differences in the Caf group with the Caf + 0.2% or Caf + 1% groups. There was no significant difference in plasma cholesterol in the Caf group compared to the C group, and there were no significant differences among the Caf, Caf + 0.2%, and Caf + 1% groups. Plasma TGs in the Caf group were significantly higher than that of the C group (*p* < 0.05), and there was a significant decrease in the Caf + 1% group compared to the Caf groups (*p* < 0.05). The LDL-C results showed that there were no significant differences between the C and Caf groups. However, the LDL-C concentration of the Caf + 1% group was significantly higher than that of the Caf group (*p* < 0.05). There were no significant differences in HDL-C concentrations or total cholesterol/HDL-C ratios among the six groups.

### 3.4. Effect of Matcha on Liver TC and TGs

**Figure 4:**
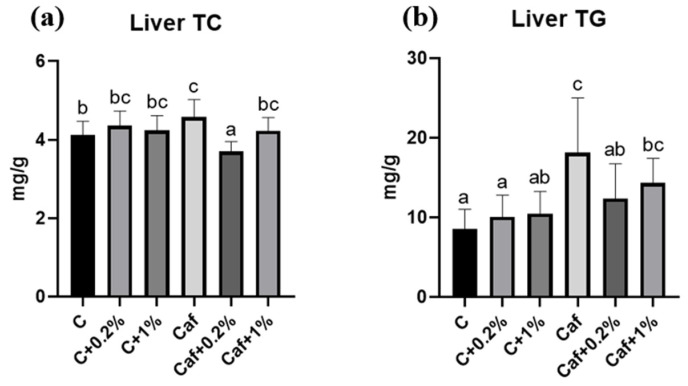
Effects of matcha on (**a**) liver total cholesterol (TC) and (**b**) triglycerides (TGs) in rats fed a cafeteria diet. Values are presented as the mean ± standard deviation (*n* = 8). ^a,b,c^ Different letters indicate a significant difference between groups at *p* < 0.05 by a one-way ANOVA with Fisher’s post hoc test. C, control diet; C + 0.2%, control diet + 0.2% matcha; C + 1%, control diet + 1% matcha; Caf, cafeteria diet; Caf + 0.2%, cafeteria diet + 0.2% matcha; Caf + 1%, cafeteria diet + 1% matcha.

As given in Figure 4, the liver TC and TG concentrations of the Caf group had significantly increased after 12 weeks of the experiment compared to the C group (*p* < 0.05). The hepatic TC and TG concentrations of the Caf + 0.2% group had significantly decreased compared to the Caf group (*p* < 0.05) (Figure 4a,b).

### 3.5. Effect of Matcha on Liver Histopathological Changes

**Figure 5:**
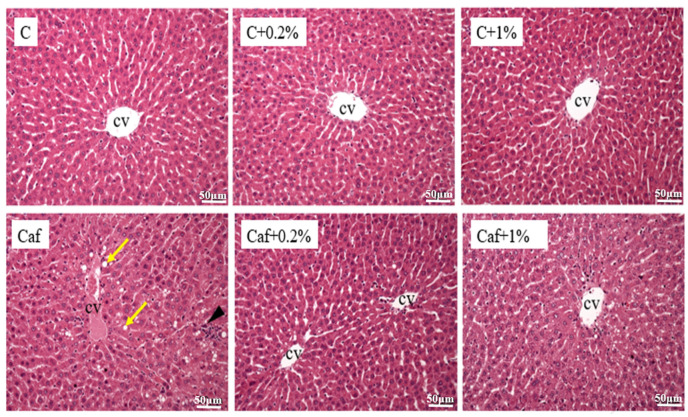
Effects of matcha on liver histopathological changes in rats fed a cafeteria diet. Hematoxylin and eosin (H&E) staining analysis of each group at 200× magnification (*n* = 5). Yellow arrows indicate macrovesicular steatosis; black arrows indicate inflammatory cell infiltration. CV, central vein. C, control diet; C + 0.2%, control diet + 0.2% matcha; C + 1%, control diet + 1% matcha; Caf, cafeteria diet; Caf + 0.2%, cafeteria diet + 0.2% matcha; Caf + 1%, cafeteria diet + 1% matcha.

The hepatic H&E staining results are presented in Figure 5, which indicated that steatosis and inflammation were evident in the Caf group, while improvements were observed in the Caf + 0.2% and Caf + 1% groups. On the other hand, the NAFLD scores were calculated and are shown in Table 3. They reveal that the steatosis score, inflammation score, and NAFLD score of the Caf group had significantly increased compared to those of the C group (*p* < 0.05). Moreover, the steatosis scores of the Caf + 0.2% and Caf + 1% groups had significantly decreased compared to that of the Caf group (*p* < 0.05).

### 3.6. Effect of Matcha on IR

**Figure 6:**
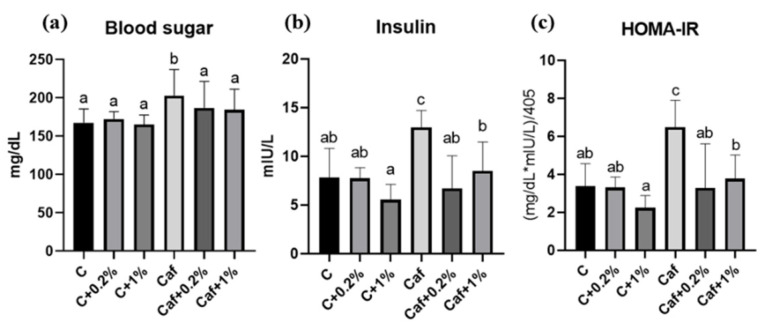
Effects of matcha on (**a**) blood sugar, (**b**) insulin, and (**c**) homeostasis assessment of insulin resistance (HOMA-IR) in rats fed a cafeteria diet. Values are presented as the mean ± standard deviation (*n* = 8). ^a,b,c^ Different letters indicate a significant difference between groups at *p* < 0.05 by a one-way ANOVA with Fisher’s post hoc test. C, control diet; C + 0.2%, control diet + 0.2% matcha; C + 1%, control diet + 1% matcha; Caf, cafeteria diet; Caf + 0.2%, cafeteria diet + 0.2% matcha; Caf + 1%, cafeteria diet + 1% matcha.

Fasting blood glucose, serum insulin, and HOMA-IR results are, respectively, shown in Figure 6a, Figure 6b, and Figure 6c. The fasting blood glucose, serum insulin, and HOMA-IR index of the Caf group were significantly higher than those of the C group (*p* < 0.05). Both the Caf + 0.2% and Caf + 1% groups had significantly lower fasting blood glucose, serum insulin, and HOMA-IR index levels compared to the Caf group (*p* < 0.05).

### 3.7. Effect of Matcha on Liver Cytokines Levels

**Figure 7:**
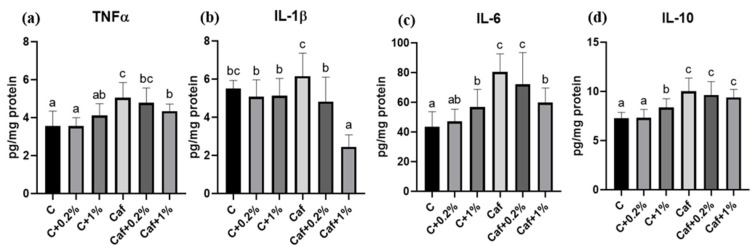
Effects of matcha on hepatic (**a**) tumor necrosis factor (TNF)-α, (**b**) interleukin (IL)-1β (**c**) IL-6, and (**d**) IL-10 in rats fed a cafeteria diet. Values are presented as the mean ± standard deviation (*n* = 8). ^a,b,c^ Different letters indicate a significant difference between groups at *p* < 0.05 by a one-way ANOVA with Fisher’s post hoc test. C, control diet; C + 0.2%, control diet + 0.2% matcha; C + 1%, control diet + 1% matcha; Caf, cafeteria diet; Caf + 0.2%, cafeteria diet + 0.2% matcha; Caf + 1%, cafeteria diet + 1% matcha.

The liver cytokine concentrations are shown in Figure 7a,d. The hepatic TNF-α and IL-6 concentrations of the Caf group were significantly higher than those of the C group (*p* < 0.05), and the levels in the Caf + 1% group were significantly lower compared to the Caf group (*p* < 0.05). Although there was no significant difference in hepatic IL-1β levels between the C and Caf groups, the hepatic IL-1β levels of the Caf + 0.2% and Caf + 1% groups were significantly lower compared to the Caf group (*p* < 0.05). As for the IL-10 results, the Caf group had a significantly increased hepatic IL-10 level compared to the C group (*p* < 0.05), but there were no significant differences among the Caf, Caf + 0.2%, and Caf + 1% groups.

### 3.8. Effect of Matcha on Plasma Adipokine Concentrations

**Figure 8:**
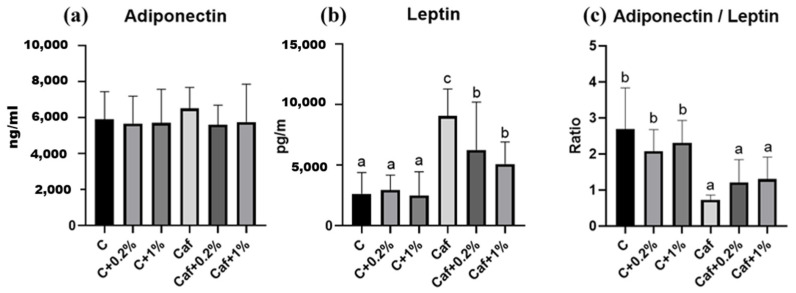
Effects of matcha on (**a**) adiponectin and (**b**) leptin levels, and (**c**) the adiponectin/leptin ratio in rats fed a cafeteria diet. Values are presented as the mean ± standard deviation (*n* = 8). ^a,b,c^ Different letters indicate a significant difference between groups at *p* < 0.05 by a one-way ANOVA with Fisher’s post hoc test. C, control diet; C + 0.2%, control diet + 0.2% matcha; C + 1%, control diet + 1% matcha; Caf, cafeteria diet; Caf + 0.2%, cafeteria diet + 0.2% matcha; Caf + 1%, cafeteria diet + 1% matcha.

Plasma adiponectin, leptin, and adiponectin/leptin ratio results are, respectively, shown in Figure 8a, Figure 8b, and Figure 8c. Although there were no significant differences in plasma adiponectin among the six groups, the plasma leptin of the Caf group was significantly higher compared to that of the C group (*p* < 0.05). Moreover, the plasma leptin levels of the Caf + 0.2% and Caf + 1% groups were significantly lower compared to that of the Caf group. The adiponectin/leptin ratio of the Caf group was significantly lower than that of the C group (*p* < 0.05), and the ratio was lower than 1.0. Although the Caf + 0.2% and Caf + 1% groups showed no significant difference compared to the Caf group, their ratios were greater than 1.0.

### 3.9. Effect of Matcha on eWAT Histopathological Staining

The eWAT H&E staining results are shown in Supplementary Figure S1. Based on those results, the size of eWATs in the Caf group was larger than that of the C group, and the size was lower in the Caf + 0.2% and Caf + 1% groups.

### 3.10. Effect of Matcha on the Gut Microbiotic Composition

**Figure 9:**
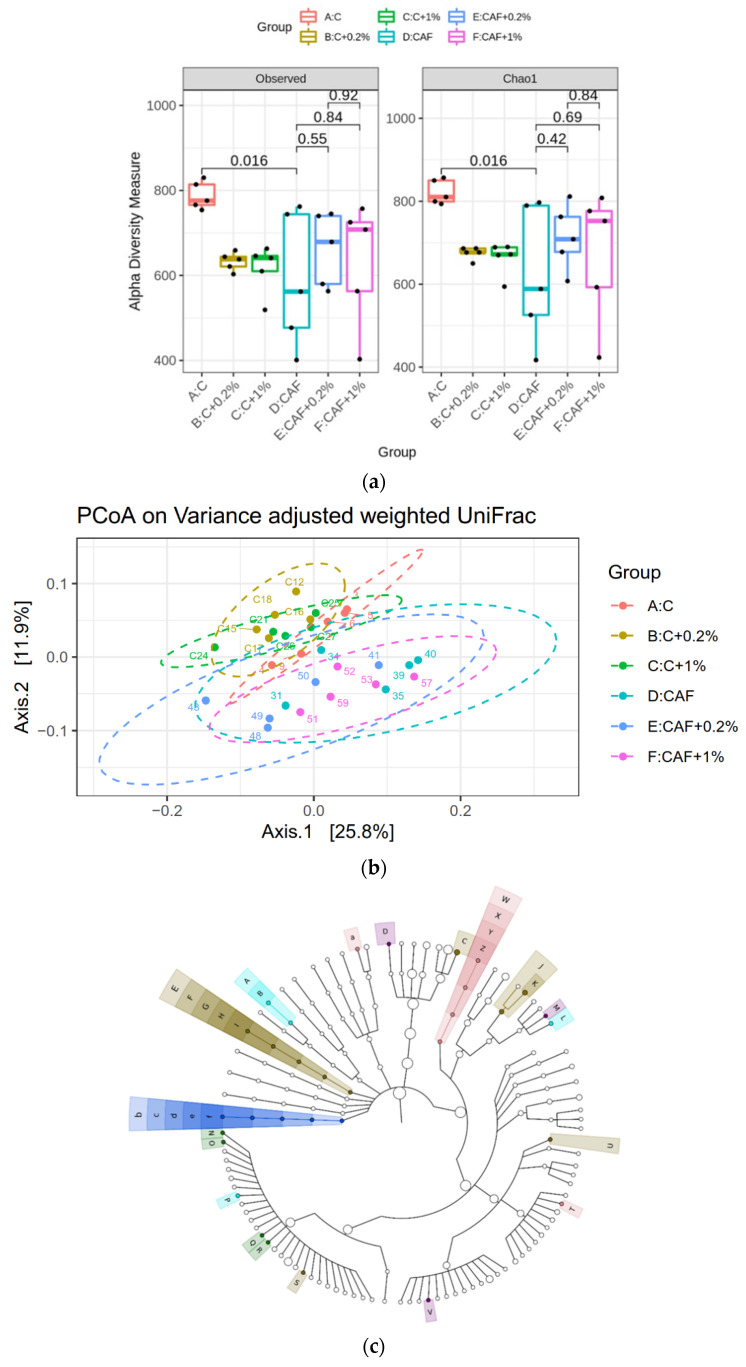

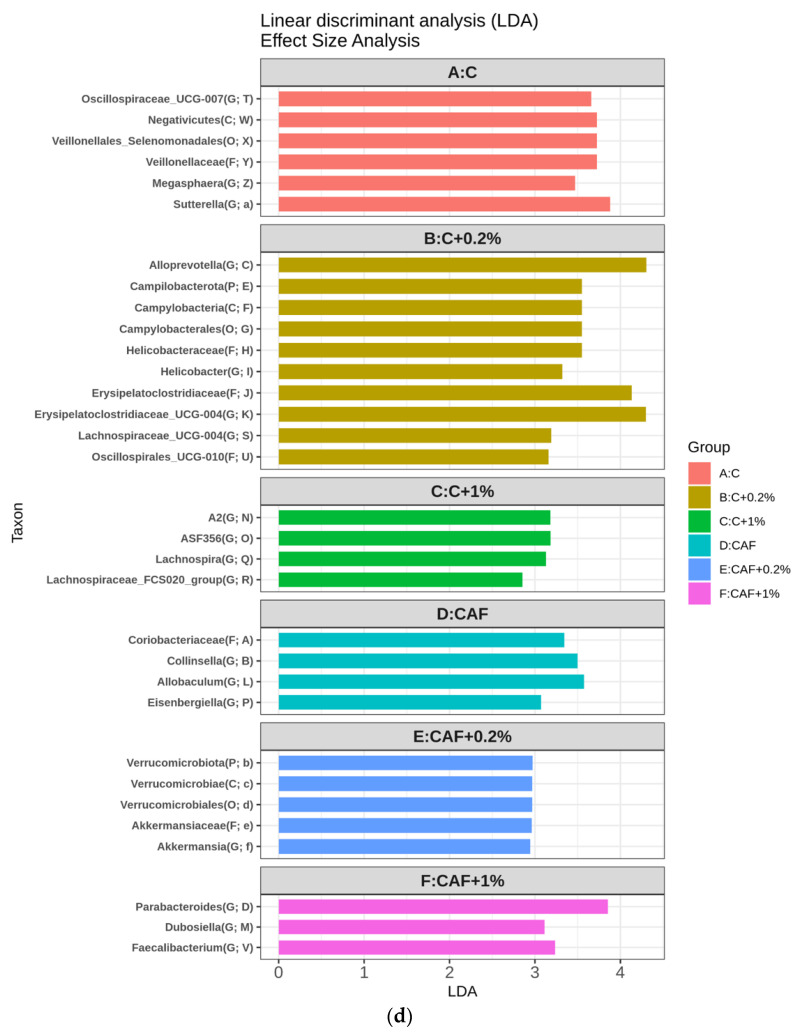
Effects of matcha on (**a**) alpha-diversity, (**b**) beta-diversity, (**c**) the linear discriminant analysis (LDA) of effect size (LEfSe), and (**d**) the LDA in rats fed a cafeteria diet.

The alpha diversity, beta diversity, linear discriminant analysis (LDA) of the effect size (LEfSe) analysis, and LDA of the gut microbiota are, respectively, shown in Figure 9a, Figure 9b, Figure 9c, and Figure 9d. As for the Observed and Chao1 alpha diversity, the microbiotic diversity in the Caf group was significantly lower than the C group. No significant difference was found in the cafeteria diet with matcha-treated groups (Figure 9a). PCoA showed a significant difference between the control-diet-fed groups and cafeteria-diet-fed groups (Figure 9b). The LDA score was ≥2 in each group as shown in Figure 9d. Significantly higher *Collinsella* together with *Allobaculum* were seen in the Caf group. On the other hand, significant increases in Akkermansiaceae and *Akkermansia* (AKK) in the Caf + 0.2% group were found. Additionally, in the Caf + 1% group, a significant increase in *Parabacteroides* and *Faecalibacterium* was observed.

## 4. Discussion

Although the Caf diet was used to investigate obesity about 40 years ago [23], in the last decade, the popularity of Caf diets has increased. The Caf diet, which is also called a “junk food diet” or “supermarket diet”, contains ultraprocessed and unhealthy but tasty foods that humans consume [13]. In addition, Sprague–Dawley and Wistar rats are the most commonly used strains in animal studies. Our Caf diet produced rats with a prediabetic syndrome such as high blood sugar and hyperinsulinemia. An abnormal lipid metabolism with elevated circulating and hepatic TGs were also observed after Caf diet feeding. In addition, hepatic proinflammatory status was found in the Caf group. All of the above physiological changes indicated that this Caf diet pattern successfully induced NAFLD in this animal model.

A previous study revealed that providing 0.1%, 0.5%, and 1% matcha powder to C57BL/6 mice for 6 weeks did not decrease their food intake [24]. Consistent with that previous study, the present data also indicated that, although matcha has a special taste, it did not affect food intake in this animal model (Figure 1). Our Caf diet induced rapid weight gain from the fourth week, followed by markedly increased visceral adiposity by the end of the study (Figure 2). Despite the fact that matcha showed no effect on BW during the study, a significantly lower eWAT and pWAT weight and smaller sizes of adipocytes (Figure 2b,c and Figure 9) were observed, which indicated a potential antiobesity effect of matcha.

Although there were no significant changes in plasma AST, ALT, TC, HDL-C, or LDL-C between the C and Caf groups, significantly higher plasma TGs, a key marker in NAFLD, were present in the Caf group. Higher plasma TGs can be attributed to the higher sugar in the Caf diet. On the other hand, 1% matcha significantly decreased the plasma TG concentration (Figure 3), which is consistent with a previous study that indicated that the bioactive chemicals of matcha could modulate hyperlipidemia [25]. Additionally, there were significantly higher hepatic TC and TG concentrations in the Caf group (Figure 4) which were consistent with the higher steatosis score in Table 3. A previous animal study revealed increased hepatic TC and TG concentrations after feeding on 18 kinds of junk food for 20 weeks [26], while our Caf diet induced hepatic lipid accumulation after only 12 weeks. On the other hand, our results showed that 0.2% matcha exhibited hepatoprotective effects by improving hepatic lipid accumulation, which is consistent with a previous study which found that matcha powder modulated steatosis under 60% high-fat-diet feeding [25]. EGCE, the most abundant compound in matcha, has the beneficial effect of improving the serum lipid profile and liver pathologic changes [27]. The matcha powder in this study was also rich in EGCG (Table 1); thus, we speculated that the hepatoprotective effect of matcha could be partially attributed to the higher EGCG content.

Metabolic dysfunction is closely associated with NAFLD, and IR is a common pathophysiological phenomenon of these two diseases [28]. IR is caused by an imbalance between energy intake and expenditure which leads to the lipolysis of adipose tissues. The increased uptake of FFAs in the liver follows high levels of circulating FFAs, which then result in hepatic lipid accumulation [29]. In this study, 12 weeks of Caf diet feeding caused significantly higher levels of blood sugar, insulin, and HOMA-IR which indicated that IR and prediabetic syndrome had occurred and may be correlated with steatosis. On the other hand, the 0.2% and 1% matcha interventions improved IR, together with a lowered lipid accumulation in the liver (Figure 4, Figure 5 and Figure 6). Although several studies have discussed that green tea extract has beneficial effects on insulin sensitivity [30,31], in this study, we first demonstrated that matcha powder has promising potential to ameliorate IR under cafeteria diet feeding.

A high-fat diet can induce liver inflammation by increasing the proinflammatory cytokines, including TNF-α, IL-1β, and IL-6, and decreasing the anti-inflammatory IL-10 cytokine, together causing systemic IR and a progression toward NASH [32,33]. In this study, the hepatic TNF-α and IL-6 concentrations significantly increased under Caf diet feeding, and these two inflammatory cytokines are involved in generating IR. TNF-α induces the c-Jun N-terminal kinase (JNK) pathway, which then inactivates the insulin receptor substrate, and IL-6 regulates the suppressor of cytokine signaling (SOCS)-1 and SOCS-3, both of which are pathways which cause IR [34]. On the other hand, we speculated that a significantly higher IL-10 in the CAF group indicated the compensation of liver function in the 12th week. Furthermore, 1% matcha (Caf + 1%) produced significantly lower TNF-α, IL-1β, and IL-6 levels compared to the CAF group, which indicated that a higher dosage of matcha may ameliorate the inflammatory response under Caf diet feeding.

Low-grade inflammation generated from adipose tissues plays a key role in obesity-related diseases, including NAFLD. Adipose tissue inflammation is affected by an imbalanced adipokine profile which impacts NAFLD by regulating both hepatic fat accumulation and IR [9]. Adiponectin is regarded as an anti-inflammatory mediator, while leptin modulates inflammation. Adiponectin is markedly decreased in NASH, atherosclerosis, and T2DM [35], while increased circulating leptin levels are usually correlated with the severity of liver disease [32]. In this study, there were no significant differences in plasma adiponectin among all groups (Figure 8), which may indicate an adaptive mechanism or an earlier development of NAFLD in our model. However, the markedly elevated plasma leptin level together with increased eWAT and pWAT weights and adipose sizes (Figure 2 and Figure 8, Supplementary Figure S1) revealed that obesity and leptin resistance occurred in our Caf diet model. On the other hand, the matcha intervention ameliorated leptin resistance and epididymal adipose cell hypertrophy, which may be correlated with lower levels of hepatic proinflammatory cytokines (Figure 7 and Figure 8, Supplementary Figure S1). Moreover, an adiponectin/leptin ratio of ≥0.5 <1.0 of the Caf group suggests a moderate-medium increased cardiometabolic risk [36].

The gut microbiota plays a key role in NAFLD. *Collinsella* is correlated with obesity and atherosclerosis, and also with a link to the fasting level of TGs [37]. In addition, *Allobaculum* increases the risk of diabetes which then progresses to NAFLD [38]. The above two genera significantly increased in the Caf group in this study, which indicated that 12 weeks of ingesting snacks and processed foods led to a microbiotic imbalance and contributed to NAFLD. On the other hand, 0.2% and 1% matcha enriched the abundance of beneficial bacteria, such as *Parabacteroides*, *Akkermansia,* and *Faecalibacterium*. *Parabacteroides* and *Akkermansia* are considered to relieve obesity, diabetes, and metabolic syndrome [39]. In addition, *Faecalibacterium* ameliorated NAFLD in a mouse study [40]. Taken together, matcha improving cafeteria-diet-induced NAFLD in a rat model may have partially been due to the regulation of the gut microbiota.

Although previous studies showed the benefits of anti-inflammation and an improvement in metabolic disorder by matcha [12,25], there are still limited data investigating the effect of matcha on NAFLD. Compared to green tea, when we drink matcha or enjoy matcha-containing snacks, we take in almost all of the ingredients of tea leaves, both the water-soluble and insoluble parts. Therefore, matcha shows greater potential health benefits than green tea [25]. In this study, both the 0.2% and 1% matcha interventions showed similar protective effects on regulating IR, lipid metabolism, and liver proinflammatory cytokines. In our animal study, 0.2% matcha powder equaled the consumption of 1.2 g of matcha powder per day for a 60 kg person, whereas 1% matcha equaled 6 g of matcha powder. In Japanese tea culture, one cup of matcha tea contains about 2 g of matcha powder, which means 0.2% matcha powder for rats equaled a half cup of matcha tea for humans. Our study first confirmed that even a half cup of matcha tea per day could ameliorate Caf-diet-induced NAFLD.

The doses of matcha employed in this animal study are translatable to clinical settings, as the low and high experimental doses correspond to the daily consumption of approximately half a cup and three cups of matcha, respectively, quantities achievable in everyday life. Nevertheless, the relatively small sample size may limit the generalizability of these findings to clinical populations, representing a notable limitation of the study. Future research could integrate dietary modifications and increased physical activity to mitigate fatty liver and obesity, thereby enhancing the translational potential of these interventions.

## 5. Conclusions

Twelve weeks of Caf diet consumption contributed to increased plasma and hepatic TG concentrations, IR, increased hepatic proinflammatory cytokines, imbalanced adipokines levels, and also dysbiosis. Combining 0.2% or 1% matcha powder decreased plasma and hepatic TG concentrations and ameliorated IR. In addition, lower hepatic proinflammatory cytokines and plasma leptin concentrations were found in the matcha group. We concluded that matcha exerted a potential hepatoprotective effect in rats with Caf-diet-induced NAFLD, which may be partially attributed to the restoration of the gut microbiota.

## Figures and Tables

**Table 1:** Total polyphenol, caffeine, and catechin contents of the matcha powder sample.

Active Compound	Content
Total polyphenols (mg GAE/g)	81.0
Caffeine (mg/g)	24.9
GC (mg/g)	1.4
EC (mg/g)	6.4
ECG (mg/g)	11.3
EGC (mg/g)	36.5
EGCG (mg/g)	50.4

Data were analyzed by SGS Taiwan (New Taipei City, Taiwan). GAE, gallic acid equivalents; GC, gallocatechin; EC, epicatechin; ECG, epicatechin gallate; EGC, epigal locatechin, EGCG, epigallocatechin gallate.

**Table 2:** Effects of matcha on body weight at 0, 4, 8, and 12 weeks of the intervention in rats fed a cafeteria (Caf) diet.

Body Weight (g)	C	C + 0.2%	C + 1%	Caf	Caf + 0.2%	Caf + 1%
0th week	293.4 ± 17.2	293.4 ± 13.0	286.1 ± 12.8	289.4 ± 11.4	289.3 ± 15.8	284.3 ± 13.8
4th week	407.4 ± 15.9 ^a^	408.8 ± 13.7 ^a^	392.8 ± 18.3 ^a^	450.6 ± 26.8 ^b^	434.8 ± 22.3 ^b^	445.6 ± 23.6 ^b^
8th week	481.0 ± 20.4 ^a^	482.4 ± 10.6 ^a^	455.1 ± 18.2 ^a^	538.5 ± 45.9 ^b^	518.9 ± 44.1 ^b^	543.1 ± 48.5 ^b^
12th week	540.3 ± 30.6 ^ab^	552.3 ± 24.9 ^ab^	521.2 ± 18.1 ^a^	610.8 ± 55.0 ^c^	584.0 ± 58.1 ^bc^	616.6 ± 59.2 ^c^

Values are presented as the mean ± standard deviation (*n* = 8). ^a,b,c^ Different letters in the same row indicate a significant difference between groups at *p* < 0.05 by a one-way ANOVA with Fisher’s post hoc test. C, control diet; C + 0.2%, control diet + 0.2% matcha; C + 1%, control diet + 1% matcha; Caf, cafeteria diet; Caf + 0.2%, cafeteria diet + 0.2% matcha; Caf + 1%, cafeteria diet + 1% matcha.

**Table 3:** Non-alcoholic fatty liver disease (NAFLD) scores of each group.

Item	C	C + 0.2%	C + 1%	Caf	Caf + 0.2%	Caf + 1%
Steatosis score	0.0 ± 0.0 ^a^	0.0 ± 0.0 ^a^	0.0 ± 0.0 ^a^	0.8 ± 0.3 ^c^	0.3 ± 0.4 ^ab^	0.2 ± 0.4 ^ab^
Inflammation score	0.2 ± 0.4 ^a^	0.2 ± 0.4 ^a^	0.8 ± 0.4 ^ab^	1.4 ± 0.9 ^b^	1.6 ± 0.5 ^b^	1.6 ± 1.1 ^b^
NAFLD activity score	0.2 ± 0.4 ^a^	0.2 ± 0.4 ^a^	0.8 ± 0.4 ^a^	2.2 ± 1.0 ^b^	1.9 ± 0.2 ^b^	1.8 ± 1.3 ^b^

Values are presented as the mean ± standard deviation (*n* = 5). ^a,b,c^ Different letters in the same row indicate a significant difference between groups at *p* < 0.05 by a one-way ANOVA with Fisher’s post hoc test. C, control diet; C + 0.2%, control diet + 0.2% matcha; C + 1%, control diet + 1% matcha; Caf, cafeteria diet; Caf + 0.2%, cafeteria diet + 0.2% matcha; Caf + 1%, cafeteria diet + 1% matcha.

## Data Availability

The datasets generated during and/or analyzed during the current study are available from the corresponding author upon reasonable request.

## Supplementary Materials

The following supporting information can be downloaded at: https://www.mdpi.com/article/10.3390/nu17193051/s1, Figure S1: Effects of matcha on histopathological changes of epididymal white adipose tissues (eWATs) in rats fed a cafeteria diet.
