# Supplementary material for: Matcha Green Tea Improves Cafeteria-Diet-Induced NAFLD by Modulating the Gut Microbiota in Rats
Source: Nutrients. 2025 Sep 24;17(19):3051. doi: 10.3390/nu17193051 (PMC12525714; doi:10.3390/nu17193051)
Supplement: Supplementary file 1 [file nutrients-17-03051-s001.zip › nutrients-3857190-supplementary.pdf]

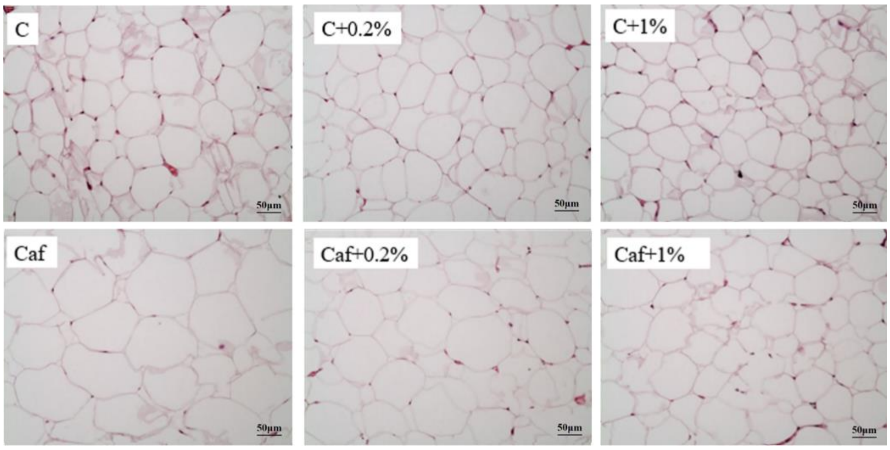

Supplementary Figure S1. Effects of matcha on histopathological changes of epididymal white adipose tissues (eWATs) in rats fed a cafeteria diet.

Hematoxylin and eosin (H&E) staining analysis of each group at 200× magnification (n = 5).
